# Case reports of primary lymphedema tarda in South Korea: Two case reports of unilateral primary lymphedema tarda in lower extremities

**DOI:** 10.1097/MD.0000000000034214

**Published:** 2023-07-07

**Authors:** Wonsik Dho, Zee Won Seo, Ju Hyun Son, Chang-Hyung Lee

**Affiliations:** a Department of Rehabilitation Medicine, Rehabilitation Hospital, Pusan National University Yangsan Hospital, Pusan National University School of Medicine, Gyeongsangnam-do, Republic of Korea; b Research Institute for Convergence of Biomedical Science and Technology, Pusan National University Yangsan Hospital, Gyeongsangnam-do, Republic of Korea.

**Keywords:** complex decongestive lymphatic physical therapy, lymphangiography, primary lymphedema tarda

## Abstract

Primary lymphedema is a form of lymphedema marked by a dysfunction of the lymphatic system without preceding medical conditions. One rare subtype of primary lymphedema, lymphedema tarda, occurs in those older than 35 years and is difficult to diagnose. This paper reports 2 cases of unilateral lymphedema tarda in the lower extremities in South Korea.

**Patient concerns:**

The 2 patients complained of worsening swelling in the lower extremity for several months without any direct surgical or traumatic history related to the inguinal or lower extremity lymphatic system.

**Diagnosis:**

Primary lymphedema tarda may be determined by ultrasonography. Other vascular or infection-origin causes were excluded from further evaluations.

**Interventions:**

To confirm primary lymphedema tarda, lymphangiography was performed. In each case, lower extremity lymphangiography indicated dermal backflow and no lymph node uptake at the inguinal node of the affected side, which was compatible with lymphedema.

**Outcomes:**

The patients reported slight improvement in the symptoms after several weeks of rehabilitation.

**Lessons:**

This paper is the first report of the unilateral primary lymphedema tarda in South Korea. Further investigations are warranted to find the related etiology of this rare disease and a multimodality regimen is needed for improvement of symptoms.

## 1. Introduction

Lymphedema is defined as the accumulation of tissue fluid resulting from impaired lymphatic drainage.^[1]^ Lymphedema is classified into primary and secondary lymphedema. Primary lymphedema is rare, affecting 1 in 100,000 individuals, while secondary lymphedema is relatively common, with an incidence of approximately 1 in 1000 Americans.^[2]^ The most common cause of secondary lymphedema worldwide is filariasis caused by Wuchereria bancrofti in endemic countries, followed by cancer-related lymphedema.^[3,4]^ Approximately 37% of women treated with gynecologic or oncologic conditions develop lymphedema, and >90% of head and neck cancer patients experience some form of lymphedema.^[5,6]^ Nevertheless, there is no standard measurement scale or poor identification of the lymphatic system worldwide. The prevalence has only been studied in the oncologic population.

Primary lymphedema is a form of lymphedema marked by a dysfunction of the lymphatic system, such as hyperplasia, hypoplasia, or aplasia of the lymphatic vessels without preceding other medical conditions.^[7]^ Primary lymphedema is associated with a dysfunction of the lymphatic system and can also develop from other vascular abnormalities, such as Klippel–Trenaunay–Weber syndrome and Turner syndrome.^[8,9]^

Primary lymphedema can be divided into 3 forms depending on the age of onset: congenital lymphedema, lymphedema praecox, and lymphedema tarda.^[10,11]^ Congenital lymphedema presents at birth or is recognized within 2 years of birth. Lymphedema precox is the most common subtype that occurs during puberty or the beginning of the third decade of life. Lymphedema tarda begins after 35 years of age, with the legs being the area most often affected.

Lymphedema tarda accounts for approximately 10% of all primary lymphedema cases.^[4]^ On the other hand, the prevalence of these rare cases has been underestimated because of the few associated studies.^[12]^ In addition, there are no reports of lymphedema tarda in South Korea. This report introduces 2 cases of unilateral lymphedema tarda in the lower extremity. Table 1 lists the demographic factors of the 2 cases of patients.

**Table 1:** Demographic factors of the 2 patients.

Variable	Case 1	Case 2
Age (year)	64	64
Sex	Female	Female
Height (cm)	147.7	160.0
Weight (kg)	63.1	50.0
BMI (kg/m^2^)	28.9	19.5
Lesion side	Left	Right
Lymphedema stage	2	2
Circumference difference (lesion – sound) (cm)		
15 cm AK	9.0	8.0
15 cm BK	4.0	3.0
ΔCSA ratio (lesion: sound)		
15 cm AK	1.93	1.95
15 cm BK	2.53	2.33
BIA ratio (lesion: sound)		
Z at 5 kHz (Ω)	0.61	0.52
Z at 1 kHz (Ω)	0.60	0.51
ECW	0.40	0.41
Biodex ratio (lesion: sound)		
EXT	1.00	0.92
FLX	1.10	0.72

AK = above the knee, BIA = bio-impedance analysis, BK = below the knee, BMI = body mass index, ECW = extracellular water, EXT = knee extension peak torque, FLX = knee flexion peak torque, Z = impedance.

## 2. Case 1

**Figure 1.**
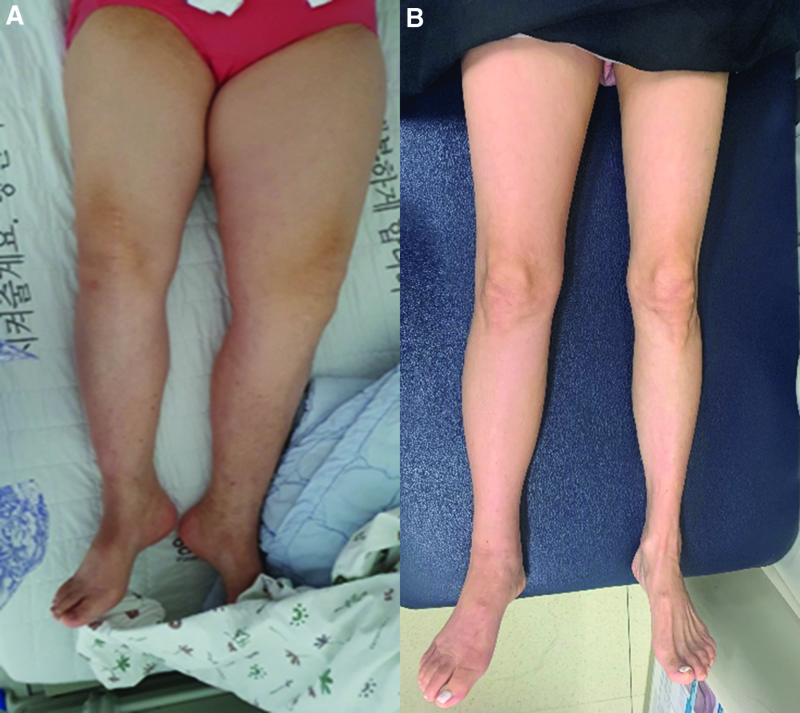
Lower extremities of (A) case 1 and (B) case 2.

A 64-year-old female visited the authors’ cancer rehabilitation clinic with a painless, progressive swelling of the left lower extremity of a 1-year duration (Fig. 1A). She was obese and her vital signs were normal. She had no direct surgical or traumatic history related to the inguinal or lower extremity lymphatic system. In addition, she had no allergies and no family history of lymphedema and had never been overseas.

On physical examinations, the left lower extremity below the inguinal line was noticeably larger than the right side. Fibrotic changes and stemmer signs were observed in the lesion side. There was no heating sensation and skin redness in the lower extremity. The leg circumferences measured at 15 cm above knee (AK) were 43 cm at the right and 52 cm at the left. The leg circumferences measured at 15 cm below knee (BK) were 32.5 cm and 36.5 cm, respectively.

**Figure 2.**
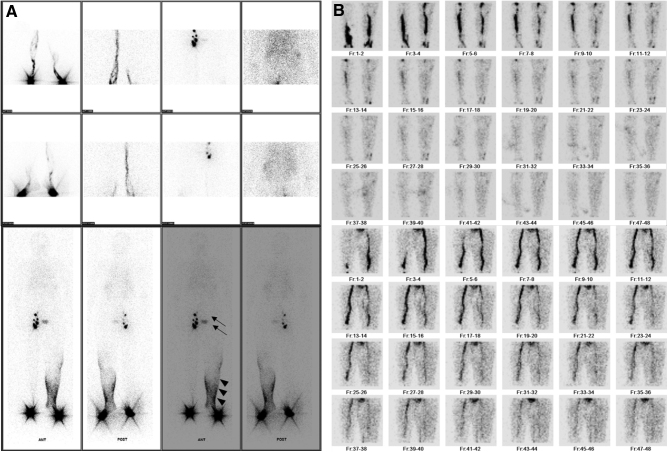
(A) RI lymphangiography confirmed lymphatic obstruction of the left lower limb. The delayed flow of the lymphatic channel of the left leg and dermal backflow is suspicious (arrowheads). The left inguinal lymph node is not found in the study (arrows). (B) Venography showed no venous obstruction in both lower extremities.

The diagnosis of lymphedema was confirmed by lymphangiography, which revealed the presence of dermal backflow and the absence of lymph node uptake at the inguinal node (Fig. 2A). Venography showed no venous obstruction (Fig. 2B). Routine laboratory data, including the markers related to renal function or tumor or parasite infections, were within the normal limits. The D-dimer was 2.65 ug/mL, which is within the upper normal limits. Cardiac evaluations, including electrocardiography and echocardiography, presented no abnormal findings.

**Figure 3.**
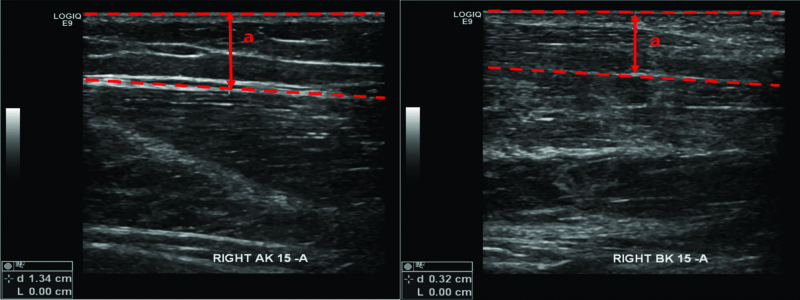
Ultrasonographic measurement of ΔCSA in the lower extremity (a: distance between skin and the fascia of the muscle). AK = above the knee, BK = below the knee, ΔCSA = cross-sectional area.

The state of lymphedema was evaluated using ultrasonographic techniques.^[13]^ Ultrasonography was used to measure the distance between the skin and the muscle fascia 3 times each at the superior, medial, inferior, and lateral aspects of the bilateral leg, at 15 cm AK and BK (Fig. 3). The amount of soft tissue in the cross-sectional area was calculated from a designed formula using the mean thicknesses on 16 sites.^[13]^ According to Lee et al, the ratio of the cross-sectional area was calculated to be 1.93 for 15cm AK and 2.53 for 15cm BK. Bioelectrical impedance analysis (BIA) using single-frequency-BIA (InBody S10; Biospace, Seoul, Republic of Korea) and isokinetic muscle strength test using an isokinetic dynamometer (System 4 Pro^TM^; Biodex Medical Systems, Inc., NY) were also performed to measure the lymphedema amount and muscle amount, respectively. Impedance ratios of lesion to sound side were measured as 0.60 at 1kHz and 0.61 at 5kHz, respectively. The extracellular water ratio was measured as 0.404. The isokinetic muscle strength ratios of lesion to sound side were measured as 1.00 at knee 60° extension and 1.10 at knee 60° flexion, respectively.

Patient received intensive rehabilitation during admission for 2 weeks. The rehabilitation programs included physiotherapy, manual lymphatic drainage, and pneumatic compression for 2 hours 5 days a week. Physiotherapy focused on strengthening the lower extremities and core muscles while wearing lower limb stockings. Manual lymphatic drainage consisted of a massage by a physical therapist pushing the patient’s leg from the distal end to the proximal direction. Pneumatic compression therapy included compression bandaging with intermittent pneumatic compression. She was asked to keep the bandage on for at least 10 hours a day.

Although the usual treatment for typical lymphedema was performed, she reported a slight improvement in the subjective symptoms, but there was no significant improvement in the circumference after treatment.

## 3. Case 2

A 64-year-old female visited the outpatient clinic with a painless, progressive swelling of the right lower extremity for 6 months (Fig. 1B). She reported that the range of the lesion aggravated gradually from the ankle to the calf and thigh. At first visit, she was not obese and her vital signs were normal. She had never consumed alcohol or smoked. She had no related risk factors including surgical, traumatic, allergic, and family history. She had been to China without an infection history.

A physical examination revealed the right lower extremity below the inguinal line to be noticeably larger than the left side. Concomitantly, fibrotic changes and stemmer signs were observed in the same region. There was no heating sensation and skin redness in the lower extremity.

**Figure 4.**
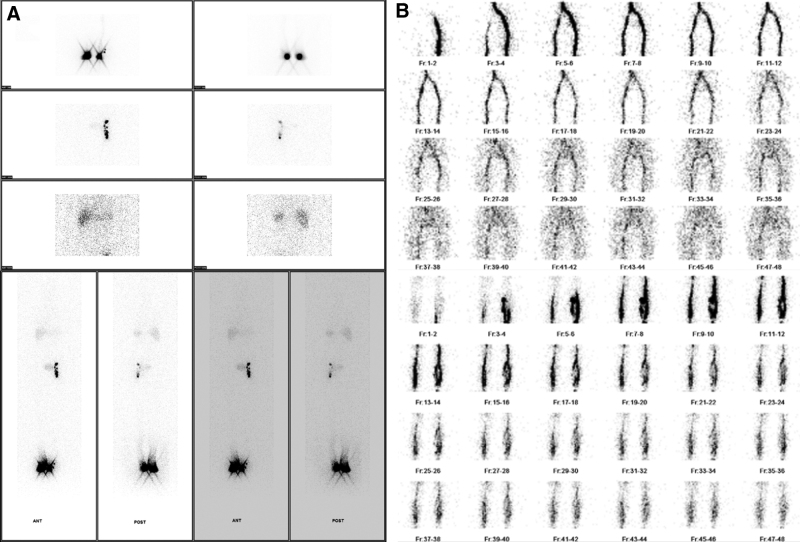
(A) Lower extremity lymphangiography confirmed the lymphatic obstruction of the right lower limb. Right inguinal lymph node activity was not found in the study (arrows). (B) Venography showed no venous obstruction in both lower extremities.

The patient underwent lower extremity lymphangiography, which revealed the absence of inguinal lymph nodes (Fig. 4A). Venography showed no venous obstruction (Fig. 4B). Other indicators for evaluating the status of the lymphedema can be confirmed by comparing with case 1 in Table 1.

The patient had received outpatient-based rehabilitation for 1 month. The same treatments as those in case 1 were administered for 1-hour twice a week. In this case, all treatments included teaching home-based exercise. As in case 1, despite intensive outpatient treatment for lymphedema, there were no significant improvements in swelling, except for her subjective improvement.

## 4. Discussion

Except for parasite infections in endemic countries, lymphedema of the lower extremities often occurs after surgery or chemotherapy in malignant diseases, such as cervical or colon cancer.^[6]^ On the other hand, it could be easy to overlook a lymphatic system dysfunction when there is no apparent related history or the presence of obesity. Although there are various measurement systems to diagnose lymphedema, there is no standard measurement protocol. Moreover, the procedures are expensive with only moderate sensitivity.

In the present cases, the patients referred to the authors’ clinic were confirmed no vascular dysfunction or history of cancer, or signs of parasite infections. In particular, filarial lymphedema was excluded because the patients did not have any typical clinical features of filarial infections, such as chronic development of lymphedema over the years or involvement of other sites like upper limbs, scrotum, and breast.^[14]^ The presence of lymphatic system dysfunction was confirmed using lymphangiography. Ultrasonography and circumference measurement were used as the degree of lymphedema, and a physical examination determined the fibrosis state. The muscle strength was measured using an isokinetic strength test, and the state of extracellular fluid collection of the lesion side was assessed by BIA. After confirming lymphedema tarda, complex decongestive lymphatic physical therapy was applied for 2 weeks of admission (1st case) or 4 weeks of outpatient follow-up (2nd case). The state of the lymphedema was improved slightly in both cases.

Even if there is no history of cancer, early diagnosis with useful diagnostic methods, such as ultrasonography or lymphangiography, should be considered to exclude primary lymphedema. Although there had been no reported etiology related to this lymphatic dysfunction, further efforts to determine the causes of this disease and continuous follow-up will be needed to exclude disease progression.

## Author contributions

**Data curation:** Zee Won Seo, Ju Hyun Son.

**Investigation:** Ju Hyun Son.

**Project administration:** Ju Hyun Son, Chang-Hyung Lee.

**Supervision:** Chang-Hyung Lee.

**Writing – original draft:** Zee Won Seo, Wonsik Dho.

**Writing – review & editing:** Wonsik Dho.
